# Editorial: Impact of cutting-edge research on legume biology- new-age technological interventions in breeding and “omics” science for sustainable and new-age climate-smart plants

**DOI:** 10.3389/fpls.2024.1405440

**Published:** 2024-05-24

**Authors:** Musa Kavas, Sagarika Mishra

**Affiliations:** ^1^ Department of Agricultural Biotechnology, Faculty of Agriculture, Ondokuz Mayıs University, Samsun, Türkiye; ^2^ Department of Science and Technology (DST) Women Scientist -A, Institute of Life Sciences, Bhubaneswar, India

**Keywords:** legume, biotic stress, abiotic stress, breeding, transcriptome, genomics, GWAS

Legumes are nutritionally and agronomically-indispensable crops in the era of food globalization. Currently, legumes have profound potential and commercial importance in the food industry owing to their applicability towards value-added food supplements. However, environmental cues such as, biotic and abiotic stress factors impose serious challenges in the overall attainment of global food security. Thereby, the improved and sustainable production of these crops necessitates continuous implementation strategies employing rigorous selective and developmental technological interventions.

This Research Topic consists of 4 original research articles highlighting the recent developments in legume biology employing the advanced omics and breeding techniques. How these integrated research findings have contributed towards the development of sustainable, nutrient-enriched, biotic and abiotic stress resilient crops have also been discussed.

Micronutrient deficiency often quoted as “hidden hunger” is detrimental to both human and animal health. Biofortification is an environmental-friendly and cost-effective agricultural strategy to enrich these trace elements in plant sources for better health benefits. Further, understanding the molecular signatures regulated by these processes will enhance our understanding in overcoming micronutrient deficiency in edible crops. In this topic, Wang et al. highlighted the agronomic importance of selenium biofortification in forage legume crop, alfalfa. Through a comprehensive and comparative transcriptomics study of non-coding RNA (ncRNA), they identified the differentially-expressed long non-coding RNAs, microRNAs, and circular RNAs during exogenous treatment of Se in alfalfa. Further, these findings enlighten us with the coordinated ncRNA-mediated regulatory mechanisms involved in improving the growth and resilience of alfalfa during Se biofortification.

Secondary metabolites are essential for plants towards defense responses against biotic and abiotic stresses, however, they are also known to have nutritional benefits for human health. In this topic, Kim et al. reportedly identified the key genetic factors regulating the biosynthesis of secondary metabolites across 50 mung bean genotypes of diverse origin and varied phenotypes along with the wild species. In this study, they employed ultra-high-performance liquid chromatography (UPLC) to identify the high functional substance contents mainly, catechin, chlorogenic acid, and neochlorogenic acid in mung bean sprouts. Furthermore, the authors suggested the regulation of functional metabolites at the transcriptional level in wild-type mung bean cultivars which can be exploited for improving the nutritional quality of cultivated mung bean sprouts through improved breeding and genetic engineering approaches.

Biotic stress factors are known to exert devastating effect on the growth and yield of crops. In this topic, Oladzad et al. employed combined approach of classic QTL mapping and QTL-based bulk segregant analysis (BSA) along with Khufu *de novo* QTL-seq aimed at the white mold disease linked meta-QTL WM2.2, previously mapped to a large genomic interval on Pv02 in common bean. These findings aimed at futuristic development of functional genetic markers for genomics-assisted MAS breeding and improvement of WM resistance in common bean.

Similarly, in this topic, Yasmin et al. studied the genetic basis of glyceollin elicitation against biotic stress, soybean cyst nematode (SCN) by employing metabolite-based genome-wide association study (mGWAS) in genetically diverse and understudied wild soybean roots. The authors employed genomic and evolutionary approaches to understand the genetic and selection basis of glyceollin elicitation necessary towards environmental stress response in legumes. Thereby, further providing a comprehensive biofortification application in related cultivable soybean cultivars.

## Author contributions

SM: Writing – original draft, Writing – review & editing. MK: Writing – original draft, Writing – review & editing.

